# Total hysterectomy versus uterine evacuation for preventing post-molar gestational trophoblastic neoplasia in patients who are at least 40 years old: a systematic review and meta-analysis

**DOI:** 10.1186/s12885-018-5168-x

**Published:** 2019-01-07

**Authors:** Peng Zhao, Yongchao Lu, Wei Huang, Baoqin Tong, Weiguo Lu

**Affiliations:** Department of Obstetrics and Gynecology, Women’s Hospital, Zhejiang University School of Medicine, No.1 Xueshi Road, Hangzhou, 310006 Zhejiang Province China

**Keywords:** Hydatidiform mole, Molar pregnancy, Gestational trophoblastic neoplasia, Total hysterectomy, Uterine evacuation

## Abstract

**Background:**

The clinical value of total hysterectomy for patients with hydatidiform mole (HM) being at least 40 years old remains highly controversial. Since the practice of hysterectomy has been applied globally for decades, there is an urgent need to perform a systematic review to assess its risks and benefits.

**Methods:**

Six electronic databases, including four English databases and one Chinese database, were searched from the inception of each database till October 6th 2017. Studies were included if they: 1) were human studies, 2) explicitly indicated exposure to hysterectomy, 3) explicitly indicated control to uterine evacuation, 4) explicitly indicated the participants were older patients with HM being at least 40 years in age, 5) compared the outcome of interest as the incidence of post-molar GTN. Two authors independently conducted the literature search, study selection, data extraction. Pooled odds ratios were analyzed using Review Manager 5.3.

**Results:**

The overall pooled effect size of total hysterectomy had a significant advantage in preventing post-molar gestational trophoblastic neoplasia over uterine evacuation with an OR of 0.19 (95% CI, 0.08–0.48; *P* = 0.0004) and a low heterogeneity (I^2^ = 21%, *P* = 0.28). Subgroup analysis and sensitivity analysis also showed similar results.

**Conclusions:**

Total hysterectomy, as compared to uterine evacuation, is a better therapeutic method for patients with HM being at least 40 years old unless fertility is still desired.

**Electronic supplementary material:**

The online version of this article (10.1186/s12885-018-5168-x) contains supplementary material, which is available to authorized users.

## Background

Hydatidiform mole (HM), including complete hydatidiform mole and partial hydatidiform mole, is perceived as the most common form of gestational trophoblastic disease. When HM advances to gestational trophoblastic neoplasia (GTN), it can pose a serious health threat. Fortunately, the transformation of GTN can be prevented by proper surgical therapeutic method.

Uterine evacuation and total hysterectomy have been the two main surgical therapeutic strategies in treating hydatidiform mole for years. Uterine evacuation, being a conservative method for fertility preservation, is the first line treatment choice for young patients. While for older patients who are 40 years old or older and no longer require fertility, total hysterectomy is preferred, as theoretically it can eliminate local invasion of the uterus hence prevent post-molar GTN. However, the scientific community has so far, not unanimously accepted the above-mentioned theory. While some studies demonstrated that total hysterectomy could potentially prevent the malignancy of HM [1, 2], others argued it cannot reduce the probability of GTN transformation and may even contribute to operative dissemination of trophoblastic tissue [3, 4]. Since the practice of hysterectomy has been applied globally for decades, there is an urgent need to perform a systematic review to assess the risks and benefits of total hysterectomy for patients with hydatidiform mole being 40 years old and above.

Therefore, we performed the current systematic review with meta-analysis to evaluate the effectiveness of total hysterectomy versus uterine evacuation in treating older patients with hydatidiform mole who are at least 40 years old.

## Methods

This systematic review and meta-analysis were carried out based on a predesigned protocol and were presented according to the guidelines of preferred reporting items for meta-analysis of observational studies in epidemiology group [5].

### Sources

Six electronic databases, including PubMed, Embase, Web of Science, Cochrane Library, ClinicalTrials.gov and China National Knowledge Infrastructure (CNKI), were searched using the controlled vocabulary and plain language from the inception of each database till October 6th 2017. Two authors (Peng Zhao and Yongchao Lu) conducted the research independently. The search strategies were developed without language restriction and provided in Additional file 1. The related articles function, Explode searching function and extensive searching function were applied to broaden the search.

After the literature search, all retrieved articles were stored in a citation manager (Endnote X7, Thomson Reuters, New York, NY, USA). In case of missing data, we contacted authors in the related field by e-mail trying to retrieve potential research material, including ongoing and unpublished studies We also hand-searched the reference lists of original research studies and previous systematic reviews concerning related topics in order to obtain more potentially eligible articles.

For non-English articles, English abstracts were used to examine if the article was related to the current study. When non-English full texts were provided, risk ratios or odds ratios (with confidence intervals) were taken into consideration to determine whether further translation would be required to obtain relevant data. Particularly, Chinese full text was retrieved and read carefully as all the authors are native Chinese.

### Study selection

The identification process was constituted of two steps which were title/abstract screening and full text review. Two authors (Peng Zhao and Yongchao Lu) conducted this procedure independently and the lists of citations included as well as those excluded were then cross-checked, and all discrepancies were solved by consulting a third author who is a senior researcher (Weiguo Lu) until consensus was reached. Two authors (Wei Huang and Baoqin Tong) independently extracted and entered the data.

Studies were included in the title/abstract screening if they: 1) were human studies, 2) explicitly indicated exposure to hysterectomy, 3) explicitly indicated control to uterine evacuation, 4) explicitly indicated the participants were older patients with hydatidiform mole being at least 40 years in age, 5) compared the outcome of interest as the incidence of post-molar GTN. Studies were excluded if they were 1) animal studies, 2) review articles, 3) case report or case series.

The evaluation of methodological quality of each study was performed using the scoring system of New Castle-Ottawa Scale (NOS) [6] for non-randomized studies and the Cochrane Risk of Bias assessment tool for randomized controlled trials. Disagreement was resolved by consultation and consensus.

Data were analysed using Review Manager (RevMan 5.3, The Nordic Cochrane Centre, Copenhagen, Denmark). Random effects models were employed based on the assumption that variation between studies exist, consequently, the interpretation of OR would be more conservative [7].

The statistical heterogeneity was assessed by calculating the Cochrane Q statistic and Higgins’s I^2^ value. Significant statistical heterogeneity was identified if the *P* value was less than 0.05. No heterogeneity was defined as an I^2^ value of 0. An I^2^ value of less than 25% indicated low heterogeneity, 25–50% moderate and greater than 50% high heterogeneity. We considered an I^2^ value of greater than or equal to 50% significant heterogeneous [8].

The subgroup analyses were performed by including participants separately based on their age and the study location. A further sensitivity analysis was conducted by excluding one study at a time, synthesizing high-quality studies in NOS assessment and switching random model to fixed model.

Publication bias was assessed subjectively by visual evaluation of funnel plots. Furthermore, Egger’s test and Begg’s test were both conducted using Stata 13.0 (Stata Corp, College Station, Texas, USA), and *P* value less than 0.05 indicated possibility of publication bias [9], in which case, the Duval and Tweedie’s trim and fill method with random effect would be performed to estimate the adjusted effect size [10].

## Results

### Study identification and selection

The details of study identification and selection were presented in Additional file 2 according to the guideline of Preferred Reporting Items for Systematic and Meta-Analyses [11].We searched five English electronic databases (PubMed, EMBASE, Web of science, Cochrane Library and ClinicalTrials.gov) and one Chinese database (CNKI). 645 records were identified from the above databases and one extra record was obtained from reference lists, which made a total of 646 articles. After removing the duplicates, we screened the remaining 571 records. 53 case reports and 50 reviews were excluded due to the article type. 6 articles mainly studying PSTT, 2 article studying the technology of ultrasound and 1 article aiming on the subject of choriocarcinoma were also excluded. 453 records were further removed by reading the title and abstract, leaving a list of 6 articles for full-text review. Consequently, 6 studies were finally included in this systematic review and meta-analysis.

### Study characteristics

The descriptive characteristics of the included studies were shown in Table 1. No randomized controlled trials were retrieved. Six cohort studies [12–17] with a total of 291 participants were included in the current meta-analysis. The full text of five studies [12–16] and the abstract of one study [17] were obtained. Two studies [12, 13] were conducted in USA, three studies [14–16] were conducted in Asia (one in Thailand and two in China) and one in Italy [17]. The participants were at least 40 years old in four studies [14–17], 40 to 49 in one study [13] and 50 or older in one study [12]. All participants were histologically diagnosed with hydatidiform mole consisting of both partial mole and complete mole [14, 16, 17] or only complete hydatidiform mole [12, 13, 15]. The case group included the group of patients who received either hysterectomy [12, 13, 16, 17] or uterine evacuation followed by hysterectomy [14, 15]. The control group comprised patients who received only uterine evacuation. The sample size ranged from 22 to 159. The follow-up period were 3–14 years in one study [14] and 3–12 years in another study [15], while for the other four studies [12, 13, 16, 17], such data were not reported. The outcome of each study was predominantly focused on the incidence of post-molar GTN. A few studies [15, 16] additionally reported other outcomes such as median time to remission, incidence of chemotherapy resistance and time interval for diagnosing GTN. Three studies [14–16] conducted comparisons of clinical characteristics between case group and control group to assess the selection bias, as a result, no selection bias were noted. However, the remaining two studies [12, 13] did not mention such comparison.

**Table 1:** Description of the included studies

Author	Study locale	Data collection	Study design	Study method	Participants size	Follow-up (years)	Outcome	Adjusted variable
You 1996 [14]	China	1978–1991	Retrospective cohort	Age:≥40 Years	17/13	3–14	Incidence of post-molar GTN	Age, gravidity, parity
				Diagnosis: HM				
Elias 2010 [12]	USA	1965–2009	Retrospective cohort	Age:≥50 Years	7/15	Not reported	Incidence of post-molar GTN	Not reported
				Diagnosis: CHM				
Elias 2012^a^ [13]	USA	1965–2010	Retrospective cohort	Age: 40–49 Years Diagnosis: CHM	6/68	Not reported	Incidence of post-molar GTN	Not reported
Lertkhachonsukl 2016 [16]	Thailand	2000–2015	Retrospective cohort	Age:≥40 Years	18/20	Not reported	Incidence of post-molar GTN, median time to remission	Age, gestational age, initial hCG level
				Diagnosis: HM				
Giorgione 2017 [17]	Italy	1994–2014	Retrospective cohort	Age:≥40 Years	12/64	Not reported	Incidence of post-molar GTN	Not reported
				Diagnosis: HM				
Zhao 2017 [15]	China	2004–2013	Retrospective cohort	Age:≥40 Years	35/124	3–12	Incidence of post-molar GTN, incidence of chemotherapy resistance, time interval for diagnosing GTN	Age, gravidity, parity, gestational age, initial hCG level, uterine enlargement, theca lutein cyst
				Diagnosis: CHM				

*HM* hydatidiform mole, *CHM* complete hydatidiform mole, *GTN* gestational trophoblastic neoplasia

Participants size were presented as hysterectomy group vs. uterine evacuation group. ^a^The data of 68 patients who fulfilled the inclusion criteria were extracted and included in the current study

### Quality assessment

The results of quality assessment were shown in Additional file 3 using NOS system. According to the protocol of NOS, a total of 9 stars were determined to assign to every study. We considered a study with a final star rating 7 or more as high quality. As a result, four studies [12, 14–16] each assigned with 7 or more stars were rated as high quality; two studies [13] with 6 stars were hence considered low quality.

In general, the included studies had some strength. For example, all of them used primary medical records to identify participants and all eligible cases were selected over a defined period of time in a defined hospital. However, there were still considerable methodological limitations pertaining to the retrospective nature. Four studies [12, 13, 16, 17] did not report the time of follow-up, therefore, we were not sure whether the surveillance period of the above-mentioned three studies were sufficient enough to identify all cases of post-molar GTN. Furthermore, there was no information about non-response rate concerning both case group and control group, which might introduce selection bias. Finally, because the included studies were conducted in different eras and locale, socioeconomic heterogeneity might be generated and it could hardly be solved by statistical methods.

### Publication bias

**Fig. 1:**
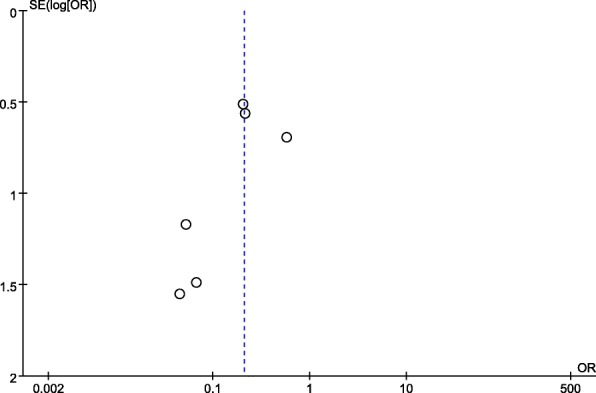
The overall funnel plot for publication bias. SE, standard error; OR, odds ratio; log, logarithm

The overall funnel plot was visually asymmetrical (Fig. 1). However, due to the limited number of eligible studies included in the current meta-analysis, we deemed the funnel plot unfeasible to test publication bias. Therefore, we further conducted Egger’s test and Begg’s test. Both tests showed no publication bias (*p* = 0.149 and *p* = 0.462, respectively).

### Meta-analysis

**Fig. 2:**
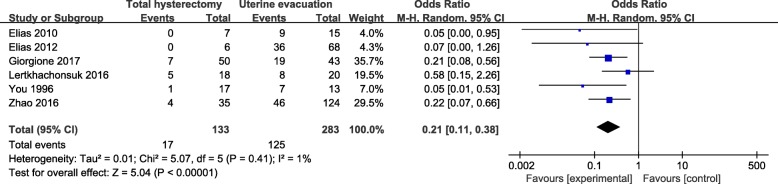
Comparison of incidence of post-molar GTN between hysterectomy group and uterine evacuation group. GTN: gestational trophoblastic neoplasia; M-H random: Mantel-Haenszel random-effects model; CI: Confidence interval

We compared the incidence of post-molar GTN in six studies. Results were presented in Fig. 2. The overall pooled effect size of total hysterectomy group had a significant advantage over uterine evacuation group with an OR of 0.21 (95% CI, 0.11–0.38; *P*<0.00001) and a low heterogeneity (I^2^ = 1%, *P* = 0.41).

**Fig. 3:**
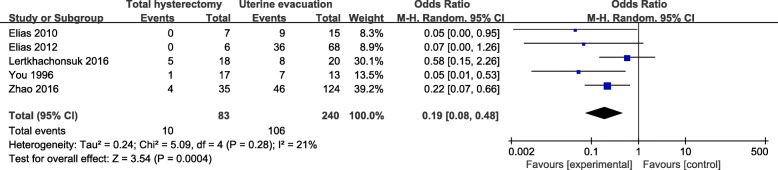
Sensitivity analysis: comparison of incidence of post-molar GTN between hysterectomy group and uterine evacuation group in high quality studies. GTN: gestational trophoblastic neoplasia; M-H random: Mantel-Haenszel random-effects model; CI: confidence interval

According to NOS system, five studies were rated as high quality. Therefore, sensitivity analysis was conducted in these studies. Results were presented in Fig. 3. Similarly, the overall pooled effect of total hysterectomy group also had a significant advantage over uterine evacuation group with an OR of 0.19 (95% CI, 0.08–0.48; *P* = 0.0004) and a low heterogeneity (I^2^ = 21%, *P* = 0.28). We further performed the sensitivity analysis by excluding one study at a time and switching random/fixed models. As a result, all of the overall pooled effects showed total hysterectomy had a significant advantage over uterine evacuation.

### Subgroup analysis

**Fig. 4:**
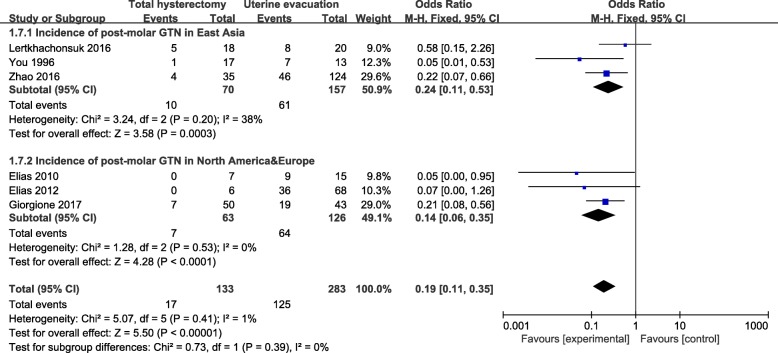
Subgroup comparison of incidence of post-molar GTN between hysterectomy group and uterine evacuation group, divided by locale. GTN: gestational trophoblastic neoplasia; M-H fixed: Mantel-Haenszel fixed-effects model; CI: confidence interval

**Fig. 5:**
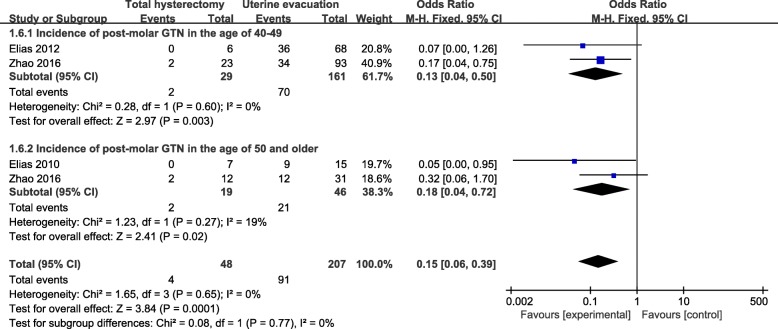
Subgroup comparison of incidence of post-molar GTN between hysterectomy group and uterine evacuation group, divided by age. GTN: gestational trophoblastic neoplasia; M-H random: Mantel-Haenszel fixed-effects model; CI: confidence interval

**Fig. 6:**
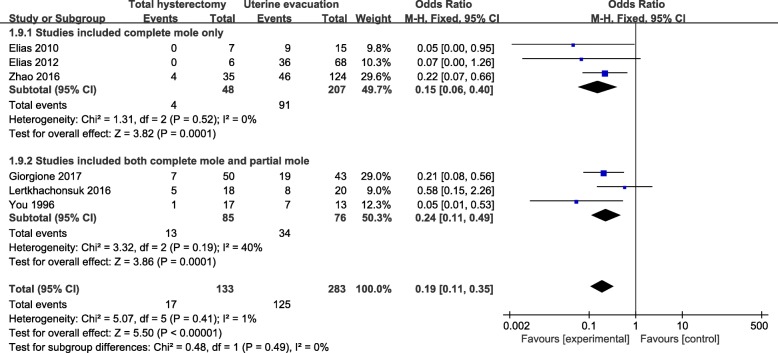
Subgroup comparison of incidence of post-molar GTN between hysterectomy group and uterine evacuation group, divided by diagnosis. GTN: gestational trophoblastic neoplasia; M-H fixed: Mantel-Haenszel fixed-effects model; CI: confidence interval

Results were presented in Fig. 4, Fig. 5 and Fig. 6. We conducted subgroup analysis on the basis of locale, age and diagnosis. In aspect of locale, we divided the participants into East Asia and North America according to the study location. We chose the term study location instead of race because there were no exact information pertaining to race in the corresponding studies, although we normally assume a study conducted in America should predominantly recruit non-Asian individuals. As a result, the overall pooled effects of total hysterectomy had significant advantages over uterine evacuation in both subgroups (Fig. 4).

Based on their age, the participants were divided into two subgroups. Subgroup 1 included patients who were in the age of 40 to 49. Subgroup 2 comprised patients who were 50 or older. Elias et al. [12, 13] reported results regarding to subgroup 1 in 2010 and subgroup 2 in 2012, respectively. Zhao et al. [15] reported results of both subgroups in one study in 2017. We extracted the data from the above-mentioned three studies and presented the outcome in Fig. 5. The other two studies cannot provide proper information for us and were therefore excluded. Consequently, the overall effects of total hysterectomy had significant advantages over uterine evacuation with low heterogeneity in both subgroups.

Based on the diagnosis, all of the included studies were divided into two subgroups. Subgroup 1 composed of three studies where the participants were diagnosed solely with complete mole, while subgroup 2 comprised the remaining three studies which recruited participants diagnosed with complete mole or partial mole. The extracted data and outcome were presented in Fig. 6. Similarly, the overall pooled effects of total hysterectomy had significant advantages over uterine evacuation in both subgroups.

## Discussion

### Main findings

The major finding of the current meta-analysis was that, in comparison with uterine evacuation, total hysterectomy could reduce the incidence of post-molar GTN in older women with HM who are at least 40 years old. And this reduction was significant with an overall OR of 0.19 (95% CI, 0.08–0.48; *P* = 0.0004) and a low heterogeneity (I^2^ = 21%, *P* = 0.28). In the light of our knowledge, no similar systematic review or meta-analysis had been reported so far.

### Strengths and limitations

Our study has several strengths. First, we have searched six dominant electronic databases without language restriction, one of which is a Chinese database considering that the incidence of HM in China is the highest among Asian countries. We also manually reviewed the reference lists of the included studies to collect more eligible articles. With these measurements taken above, we were able to conduct extensive research to produce convincing results. Second, we performed both Egger’s test and Begg’s test to detect publication bias, which is more reliable than visual observation of funnel plots. Finally, this systematic review and meta-analysis were carried out according to a predesigned rigorous protocol.

Nonetheless, the potential limitations of the current study should be recognized as well. As the included studies were all observational retrospective, unknown selection bias might be introduced and it can hardly be detected by statistical methods. Moreover, the limited numbers of patients also lowered the overall study quality, which in turn weakened the results of meta-analyses. Therefore, the results of the current study should be deliberately interpreted.

### Interpretation

There have been controversies since total hysterectomy was first introduced in 1966 to treat older patients with HM [18]. Concerns were raised that total hysterectomy could not be able to prevent post-molar GTN and may even promote vascular dissemination of trophoblastic tissue due to the surgical procedure involved hysterectomy [2, 15, 16, 19–22]. Meanwhile, the liberal performance of total hysterectomy was also favoured with the assumption that it could prevent uterine local invasion from metastasizing [6, 7, 14, 18, 23–25]. However, no general consensus has been reached so far.

It is common sense that hysterectomy can only be performed in older patients who have completed their families. Therefore, two major questions must be answered in order to solve the above-mentioned debate. 1) Is age proportional to the incidence of post-molar GTN, if so, what is the cut-off value in age? 2) Is total hysterectomy superior to uterine evacuation in treating molar pregnancy for this age-specific group of patients?

It has been well documented that the incidence of post-molar GTN increased with advanced maternal age [24, 25]. Tow, Tsuji and Tsukmoto et al. [21, 22, 26] reported respectively that 37% of women older than 40 and 56% older than 50, developed post-molar GTN as compared to a lower 15–20% in young patients. Therefore, it can be deduced that maternal age is proportionately associated with post-molar GTN, and the incidence of GTN is significantly higher when patients reach the age of 40 and older, based on which we defined 40 years old as the cut-off value. After researching the major electronic databases, we retrieved 6 studies aiming to patients with HM who are at least 40 years old. Three studies [12, 14, 15] showed that hysterectomy had better performances in preventing post-molar GTN, while three studies [13, 16, 17] showed otherwise. All of the six studies were included in our study and meta-analysis revealed that total hysterectomy, compared with uterine evacuation, significantly decreased the probability of post-molar GTN, indicating that total hysterectomy had advantages in treating older patients who had completed childbearing. Additionally, in order to confirm the above finding, subgroup analysis was conducted by patients’ age, study location and diagnosis. As a result, all of the subgroup analyses showed that total hysterectomy had an advantage in reducing the incidence of post-molar GTN over uterine evacuation. Finally, Egger’s test and Begg’s test were also conducted and no publication bias was detected. Taking all the above results together, we infer that total hysterectomy outperforms uterine evacuation in preventing post-molar GTN for patients who are 40 years old or above.

It is worth mentioning that we included two studies [16, 17] which were not in favour of total hysterectomy. In one study [17], a total of 76 patients with HM who were over 40 years old were included. The incidence of post-molar GTN were 58 and 30%, respectively (total hysterectomy versus uterine evacuation, *P* = 0.094). In the other study [16], 38 older patients with HM were included, as a result, post-molar GTN developed in 40% of patients in evacuation group and 27.8% in hysterectomy group (*p* > 0.05). The authors of both study concluded that primary hysterectomy might not be able to reduce the incidence of GTN. One may argue that the above-mentioned studies included patients who were diagnosed with invasive HM in the total hysterectomy group, which could increase the probability of post-molar GTN and seriously weaken the confidence of the results, hence should be excluded. However, in our opinion, invasive mole can mostly be detected in hysterectomy specimens, but is difficult to diagnose from evacuation specimens where myometrium tissue cannot be obtained, which is a situation of real-world studies. In fact, invasive mole may be one of the increased risks of malignancy in older patients. Excluding the two studies would threaten the credibility of the current meta-analysis.

## Conclusions

We conclude that hysterectomy, as compared to uterine evacuation, is a better therapeutic method for patients with HM who are at least 40 years old. We therefore, suggest total hysterectomy could be recommended for this age-specific group of patients unless fertility is still desired. To further confirm our findings, a multi-center randomized controlled trial should be performed.

## Additional files

Additional file 1: Search strategy. (DOC 20 kb)

Additional file 2: Quality assessment. (DOC 20 kb)

Additional file 3: PRISMA Flow Diagram. (DOC 26 kb)

## Abbreviations

CNKI: China National Knowledge Infrastructure
GTN: Gestational trophoblastic neoplasia
HM: Hydatidiform mole
NOS: New Castle-Ottawa Scale
