# Supplementary material for: Total hysterectomy versus uterine evacuation for preventing post-molar gestational trophoblastic neoplasia in patients who are at least 40 years old: a systematic review and meta-analysis
Source: BMC Cancer. 2019 Jan 7;19:13. doi: 10.1186/s12885-018-5168-x (PMC6322260; doi:10.1186/s12885-018-5168-x)
Supplement: Supplementary file 1 — Search strategy. (DOC 20 kb) [file 12885_2018_5168_MOESM1_ESM.doc]

Search strategies

The following search strategies were developed according to the framework of PICOS. Amendments were made to broaden the search when necessary.

**Pubmed:**

(((((((((((hydatidiform mole[MeSH Terms]) OR molar pregnanc*[Text Word]) OR hydatidiform mole*[Text Word]) OR hydatidiform tumor[Text Word]) OR hydatidiform tumour[Text Word]) OR complete mole*[Text Word]) OR hydatid mole*[Text Word]) OR mola hydatidosa[Text Word])) AND ((((hysterectomy[MeSH Terms]) OR hysterectomy[Text Word]) OR uterectomy[Text Word]) OR panhysterectomy[Text Word])) AND ((((((((dilatation and curettage[MeSH Terms])) OR (Curettage and Dilatation[MeSH Terms])) OR Curettage[MeSH Terms]) OR dilatation[Text Word]) OR dilation[Text Word]) OR Curettage[Text Word]) OR evacuation[Text Word])) AND (((((((((gestational trophoblastic disease[MeSH Terms]) OR gestational trophoblastic neoplasia[MeSH Terms]) OR gestational trophoblastic neoplasm[MeSH Terms]) OR gestational trophoblastic neoplasms[MeSH Terms]) OR gestational trophoblastic diseases[MeSH Terms]) OR persistent[Text Word]) OR trophoblastic[Text Word]) OR metastatic[Text Word]) OR invasive[Text Word])

**EMBASE**

#9 #1 AND #6 AND #7 AND #8

#8 'trophoblastic tumor'/exp OR 'trophoblastic tumor' AND [embase]/lim

#7 'hysterectomy'/exp OR 'hysterectomy' AND [embase]/lim

#6 #2 OR #3 OR #4 OR #5

#5 'suction curettage'/exp OR 'suction curettage' AND [embase]/lim

#4 'evacuation'/exp OR 'evacuation' AND [embase]/lim

#3 'dilatation and curettage'/exp OR 'dilatation and curettage' AND [embase]/lim

#2 'dilation and evacuation'/exp OR 'dilation and evacuation' AND [embase]/lim

#1 'hydatidiform mole'/exp OR 'hydatidiform mole' AND [embase]/lim

Each step was conducted with Embase mapping options of “Search as broadly as possible” and Sources of “EMBASE”.

**Web of science**

1.TS: (molar pregnanc*) OR TS: (hydatidiform mole*) OR TS: (hydatidiform tumor) OR TS: (hydatidiform tumour) OR TS: (complete mole*) OR TS: (hydatid mole*)

2.TS: (hysterectomy) OR TS: (uterectomy) OR TS: (panhysterectomy)

3.TS: (Curettage) OR TS: (dilatation) OR TS: (dilation) OR TS: (evacuation)

4.TS: (gestational trophoblastic disease) OR TS: (gestational trophoblastic neoplasia) OR TS: (gestational trophoblastic neoplasm) OR TS: (gestational trophoblastic neoplasms) OR TS: (gestational trophoblastic diseases) OR TS: (persistent) OR TS: (trophoblastic) OR TS: (metastatic) OR TS: (invasive)

5.#4AND #3 AND #2 AND #1

Indexes=SCI-EXPANDED, SSCI, A&HCI, CPCI-S, CPCI-SSH, BKCI-S, BKCI-SSH, ESCI, CCR-EXPANDED, IC Timespan=All years

**Cochrane Central Register of Controlled Trials**

#1

"hydatidiform mole":ti,ab,kw or "hydatidiform mole" (Word variations have been searched)

#2

"hysterectomy":ti,ab,kw or "hysterectomy" (Word variations have been searched)

#3

"D and C":ti,ab,kw or "D and C" or "curettage":ti,ab,kw or "curettage" or "dilatation and curettage":ti,ab,kw (Word variations have been searched)

#4

"gestational trophoblastic neoplasia":ti,ab,kw or "gestational trophoblastic neoplasia" (Word variations have been searched)

#5

#1 and #2 and #3 and #4

**China National Knowledge Infrastructure (CNKI)**

(FT=invasive mole or FT=malignant mole or FT=malignancy or FT=choriocarcinoma ) and (FT=hysterectomy or FT=uterine operation) and (FT=uterine evacuation or FT=uterine apiration) and ( FT=molar pregnancy or FT=gestational trophoblastic disease)

All of the search terms were translated from Chinese terms.
