# Supplementary material for: Total hysterectomy versus uterine evacuation for preventing post-molar gestational trophoblastic neoplasia in patients who are at least 40 years old: a systematic review and meta-analysis
Source: BMC Cancer. 2019 Jan 7;19:13. doi: 10.1186/s12885-018-5168-x (PMC6322260; doi:10.1186/s12885-018-5168-x)
Supplement: Supplementary file 2 — Quality assessment. (DOC 20 kb) [file 12885_2018_5168_MOESM2_ESM.doc]

**PRISMA Flow Diagram**

**Screening**

**Included**

**Eligibility**

**Identification**

**Records identified through database searching**
PubMed: 122, EMBASE: 103, Web of science: 33, CNKI: 384, Cochrane Library:3, ClinicalTrials: 0

**(n = 645)**

**Additional records identified through other sources
(n =1)**

**Records after duplicates removed: 75
(n =571)**

**Records screened
(n = 571)**

**Records excluded**

Case report: 53, Review: 50, Ultrasonography: 2, PSTT: 6,

Choriocarcinoma:1
Title and abstract excluded: 453

**(n = 564)**

**Full-text articles assessed for eligibility
(n = 6)**

**Full-text articles excluded, with reasons**

**(n = 0)**

**Studies included in quantitative synthesis (meta-analysis)
(n =6 )**
