# Supplementary material for: Total hysterectomy versus uterine evacuation for preventing post-molar gestational trophoblastic neoplasia in patients who are at least 40 years old: a systematic review and meta-analysis
Source: BMC Cancer. 2019 Jan 7;19:13. doi: 10.1186/s12885-018-5168-x (PMC6322260; doi:10.1186/s12885-018-5168-x)
Supplement: Supplementary file 3 — PRISMA Flow Diagram. (DOC 26 kb) [file 12885_2018_5168_MOESM3_ESM.doc]

Quality assessment

| **Study** | **Representativeness of the exposed cohort** | **Selection of the non exposed cohort** | **Ascertainment of exposure** | **Demonstration that outcome of interest was not present at start of study** | **Comparability of cohorts on the basis of the design or analysis** | **Assessment of outcome** | **Was follow-up long enough for outcomes to occur** | **Adequacy of follow up of cohorts** |
| --- | --- | --- | --- | --- | --- | --- | --- | --- |
| **You 1996** | ★ | ★ | ★ | ★ | ★ | ★ | ★ | ★ |
| **Elias 2010** | ★ | ★ | ★ | ★ | ★ | ★ |  | ★ |
| **Elias 2012** | ★ | ★ | ★ | ★ | ★ | ★ |  | ★ |
| **Lertkhachonsukl 2016** | ★ | ★ | ★ | ★ |  | ★ |  | ★ |
| **Giorgione 2017** | ★ | ★ | ★ | ★ |  | ★ |  | ★ |
| **Zhao 2017** | ★ | ★ | ★ | ★ | ★ | ★ | ★ | ★ |

★: Indicates good quality in the domain
